# Impact of Differentiated Service Delivery Models on Quality of Life among People living with HIV in Uganda – A Quasi-Experimental Study

**DOI:** 10.21203/rs.3.rs-5443965/v1

**Published:** 2024-12-17

**Authors:** Benson Nasasira, Grace Banturaki, Nelson Kalema, Joseph Musaazi, Aidah Nanvuma, Stephen Okoboi, Nancy Kiarie, Joash Ntenga Moitui, Damazo Kadengye, Jonathan Izudi, Barbara Castelnuovo

**Affiliations:** Infectious Diseases Institute Makerere university of health sciences; Infectious Diseases Institute Makerere university of health sciences; Infectious Diseases Institute Makerere university of health sciences; Infectious Diseases Institute Makerere university of health sciences; Infectious Diseases Institute Makerere university of health sciences; Infectious Diseases Institute Makerere university of health sciences; African Population and Health Research Center; African Population and Health Research Center; African Population and Health Research Center; African Population and Health Research Center; Infectious Diseases Institute Makerere university of health sciences

**Keywords:** HIV Infections, Delivery of Health Care, Antiretroviral Therapy, Quality of Life, Health Services Accessibility

## Abstract

**Background:**

Differentiated service delivery (DSD) models in resource-limited settings have reduced strain on health services and improved client experience, retention and viral suppression, but little is known about the impact of HIV DSD models on quality of life (QoL), which is essential for optimizing person-centered care. This study assessed the impact of DSD models on QoL, loss to follow-up (LTFU), and mortality among persons living with HIV (PLHIV) on ART over time at a large urban HIV clinic in Uganda.

**Methods:**

We analyzed records of 1,000 PLHIV who had been on ART for 10 years and followed up for eight years, starting in 2014 or 2015 at the Infectious Diseases Institute clinic in Kampala, Uganda. The primary outcome, QoL, was assessed using an adapted Medical Outcomes Study (MOS-HIV) tool. Secondary outcomes included sustained viral suppression (< 200 copies/mL), all-cause mortality, and loss to follow-up or LTFU (missing clinic visits for ≥ 3 months). Outcomes were compared across three DSD models—fast-track drug refill (FTDR), facility-based groups (FBG), and a composite model combining FTDR and FBG against the facility-based individual management (FBIM), the standard of care (SOC). Inverse probability treatment weighting was used to achieve comparability in measured covariates across the DSD models followed by mixed effects modeling. Robustness of results was checked using G-computation analysis.

**Results:**

Of 1,000 records for PLHIV, 980 were analyzed. 62% were female and 95% virally suppressed at baseline. After eight years of follow-up, participants on DSD models had higher QoL (90.4% vs 89.1%; weighted mean ratio 3.66, 95% CI 2.10–6.37, p-value < 0.001), better sustained viral suppression, lower mortality, and reduced LTFU rates compared to SOC.

**Conclusion:**

These findings support the broader adoption of DSD models in delivering ART across HIV programs to enhance the QoL and clinical outcomes among PLHIV.

## Background

Antiretroviral therapy (ART) significantly improved the life expectancy of people living with HIV (PLHIV) (1). Improved access to ART especially in the Test and Treat era substantially increased the number of PLHIV enrolled on ART potentially impacting the quality of care. To optimize ART delivery and patient-centered care, the 2016 World Health Organization (WHO) guidelines recommended Differentiated Service Delivery (DSD) models, a care approach tailored to the unique needs and preferences of PLHIV (2).

DSD models adapt HIV care based on clinical stability, patient preferences, and local context (3). Whereas Uganda adopted and implemented both facility and community-based DSD models since 2016 (Additional file 1 Figure S1), current evidence among PLHIV suggests a preference for facility-based models (4, 5). Costs, viral load suppression, and retention rates are comparable between DSD and standard health facility-based care, suggesting DSD models offer an alternative ART delivery approach without compromising the quality of care (6). A systematic review reports high retention in care and viral suppression rates associated with DSD models in sub-Saharan Africa (7). Similarly, Tsondai (2017) observed similar outcomes among PLHIV in an ART adherence club (a form of the DSD model) in South Africa (8). A meta-analysis further confirmed improvements in retention and viral suppression among PLHIV receiving care through DSD models (9).

While several studies demonstrate that DSD models improve retention and viral load suppression, there is a paucity of data on their impact on the quality of life (QoL) of PLHIV. Assessing QoL is essential for understanding the broader impacts of DSD models on individuals beyond addressing HIV clinical symptoms. This ensures continued delivery of holistic, person-centered care that addresses both medical and psychosocial needs (10). Therefore, we evaluated the impact of facility-based DSD models on QoL among PLHIV in Uganda, including whether the QoL differed by the duration of care under each model. We also assessed the impact of DSD models on sustained viral load suppression, retention in care, and mortality. Our aim is informed by a theory of change postulating that the implementation of DSD models leads to optimized HIV care resulting in improved health outcomes, namely viral load suppression, mortality, and loss to follow-up and ultimately improved QoL. We hypothesized there would be no differences in the overall QoL between PLHIV enrolled in the various health facility-based DSD models.

Evidence from this study would inform policymakers and HIV program managers about the performance of the DSD models and provide the evidence base for optimizing or sustaining health outcomes for PLHIV under the DSD models in Uganda and similar settings in sub-Saharan Africa.

## Methods

### Study setting, study design, and population

We employed a quasi-experimental design using routine patient-level observational data retrieved retrospectively from a cohort of PLHIV receiving HIV care at the Infectious Diseases Institute (IDI) HIV clinic. The clinic is located within the Mulago National Referral Hospital in Kampala, the capital of Uganda. As a center of excellence providing HIV prevention and care services, the clinic serves approximately 7,500 PLHIV, 300 of whom have complex medical conditions requiring specialized care (11). Between May 2014 and September 2015, the Antiretroviral therapy Long-Term (ALT) cohort enrolled 1,000 conveniently sampled PLHIV aged ≥ 18 years who had been on ART for 10 years and followed-up once every 12 months. The rest of the clinic visits were conducted per the clinic’s standard protocols. Further information about this cohort was published elsewhere (12). For this quasi-experimental study, we considered all PLHIV who had atleast one follow up visit in the main study to enable assessment of quality of life. As a requirement for the primary study, all participants were enrolled in facility-based DSD models. At the time of data extraction for this analysis, participants were in their 8th year of follow-up.

### Implementation of DSD models in HIV care in Uganda

Uganda’s approach to the implementation of DSD includes both facility-based and community-based approaches. The Ministry of Health’s Consolidated HIV Guidelines describes five major models: three facility-based models—Facility-Based Individual Management (FBIM), Facility-Based Group (FBG), and Fast-Track Drug Refill (FTDR)—and two community-based models—Community Drug Distribution Points (CDDP) and Community Client-Led ART Delivery (CCLAD). (Additional file 1 Figure S1). Until the year 2022, PLHIV were enrolled on DSD models based on clinical presentation—stable vs. unstable/complex as shown in Additional file 1 Figure S2. Health workers could consider additional factors such as non-communicable diseases and psychosocial support when assessing stability criteria. Based on this comprehensive assessment, PLHIV were matched with the most appropriate DSM model (Additional file 1 Figure S3), with those on stable models given longer ART refills of 3–6 months.

### Data variables and extraction

For this analysis, we retrospectively extracted data from the Integrated Clinic Enterprise Application (ICEA), an in-house electronic medical database (14). Variables included socio-demographic factors like age, sex, and marital status, and economic factors like income and employment status. Clinical factors included the WHO clinical stage, presence of opportunistic infections, CD4 cell count, viral load, anthropometric measurements (height and weight), follow-up status (active, lost to follow-up, transfer, or died), alcohol and tobacco use, quality of life, and DSD model.

### Intervention description

The intervention group comprised PLHIV who had been enrolled in any of the three facility-based DSD models for ≥ 6 consecutive months. The models included Fast Track Drug Refill (FTDR), Facility Based Group (FBG), and a switch between FTDR and FBG (FTDR + FBG). The comparison group consisted of PLHIV who had been managed under the Facility Based Individual Management (FBIM) model for a minimum of six consecutive months. The FBIM model approximates the standard of care delivery model.

### Outcomes definition

#### Quality of life definition

The primary outcome of this study was the mean QoL score, assessed at the ALT cohort baseline and during annual follow-up visits. QoL was measured using a 10-item questionnaire, nine of which were scored based on a four-point Likert scale, while one was based on a five-point Likert scale. These questions were designed to evaluate QoL across eight domains of the HIV version of the Medical Outcomes Study (MOS-HIV) tool (15), which include: general health, role function, physical health, psychological health, social relations, physical activity, mental health, Cognitive functioning, and vitality/fatigue. The MOS-HIV tool is reliable for measuring QoL among PLHIV, and has been validated in Uganda (16).

The specific QoL items included: 1) presence of sickness symptoms (none to severe); 2) the extent to which health status interfered with work, housework, or school activities (none to all the time); 3) The extent to which illness interfered with social activities (never to all the time); 4) the energy levels to complete daily tasks (always to never); 5) the highest level of physical activity the respondent could complete (vigorous to none); 6) feelings of fatigue (none to all the time); 7) difficulty remembering things (none to all the time); 8) feeling calm or peaceful (all the time to rarely); 9) feeling happy (all the time to rarely); and, 10) feeling sad or depressed (All the time to rarely).

Additionally, participants were asked to rate their overall well-being on a three-point scale (excellent, moderate, or poor). All item responses were reverse-coded before analysis, as the original coding reflected negative directions.

The secondary outcomes included:

1. **Sustained viral suppression:** This was defined as viral load < 200 copies/µL over the entire period of observation and was measured per the current Uganda Ministry of Health (MoH) guidelines (17). Viral suppression was additionally considered at < 1000 copies/mL threshold per the MoH guidelines from the inception of the viral load monitoring policy in Uganda in 2015/2016 until August 2023 (17), the period in which most of these data were accrued. For this outcome, we only included patients who were virologically suppressed at enrollment into the ALT cohort.
2. **All-cause mortality:** This was defined as death from any cause after enrollment in the cohort, measured on a binary scale (dead vs. alive).
3. **Loss to follow-up (LTFU):** This was defined based on the Uganda MoH definition during the period when data were accrued, as no clinic visit for 3 months after the latest scheduled clinic visit appointment (13).

#### Statistical analysis

Data were managed and analyzed using Stata version 17.0 and R version 4.3.3. Baseline characteristics between the intervention and comparison groups were compared using the Pearson Chi-square or Fisher’s exact tests for categorical variables. The Kruskal-Wallis test was used to compare median differences for skewed continuous variables.

QoL scores were calculated by summing scores of the individual 10-question items at each time point, converting the total to a 100% point scale. QoL scores were summarized using means and standard deviations at each study visit and presented using line graphs to show trends. Linear regression models with a squared term for time covariate were used to test for the presence of linear trends in QoL. The normality of residuals was assessed using normal plots and quantile-quantile (Q-Q) plots.

The inverse probability treatment weighting (IPTW) was used to ensure covariate balance across comparison groups and a weighted mixed effects regression model was fitted to estimate the effect of DSD models on the study outcomes. The IPTW and mixed effects model accounted for time-updated exposures, outcomes, and some covariates to avoid over-adjustment bias (18).

For the IPTW, propensity scores were calculated using a generalized linear mixed effects logistic regression model to estimate the probability of being on the DSD model, with covariates selection based on literature and subject knowledge (Additional file 1 Table S2). A Directed Acyclic Graph (DAG) informed the covariate selection. Covariates in the propensity score model included the baseline age, sex, employment status, household monthly income, WHO stage, body mass index, baseline viral load, baseline log CD4 count, baseline QoL score, having a partner, tobacco use, and alcohol use. Covariate balance was checked using standardized mean differences (SMD), with SMD < 0.1 indicating balance (Additional File 1 Table S1). Standardized IPTW weights were estimated as the ratio of unadjusted exposure probabilities to propensity scores, and the weighted mixed effects model was fitted to estimate the impact of DSD models on QoL. Sensitivity analyses involved refitting models using conventional covariate adjustments and G-computation methods, with results considered robust if they remained consistent. We tested whether differences in QoL between comparison groups varied over time (parallel assumption) by fitting an interaction term between covariates for treatment groups and time.

The weighted Cox Proportion Hazard model (Cox PH) was used to estimate the impact of DSD models on time-to-event outcomes—time to all-cause mortality or LTFU from enrollment to event or censor on December 31, 2023. Finally, a weighted logistic regression model was used to estimate the impact of DSD models on sustained viral suppression. All model assumptions were checked and validated before choosing final models for each outcome. Subgroup analyses by gender, age, and TB status were conducted for the primary outcome.

## Results

The analysis included 980 participants from the initial cohort of 1,000 participants, after excluding 20 participants who did not have any cohort follow-up visits. The majority of participants were female (61.8%) with a median age of 45 years (IQR 40, 51) years. Most participants (81.3%) were employed, with 55.4% reporting a monthly household income above 30 United States dollars. The median BMI was 23.0 kg/m^2^ (IQR 20.0–26.0) and the median baseline CD4 count was 537 cells/uL (IQR 398–715). Detailed baseline characteristics are presented in Table 1.

### Trends in mean quality of life over 8 years of follow-up

**Figure 1:**
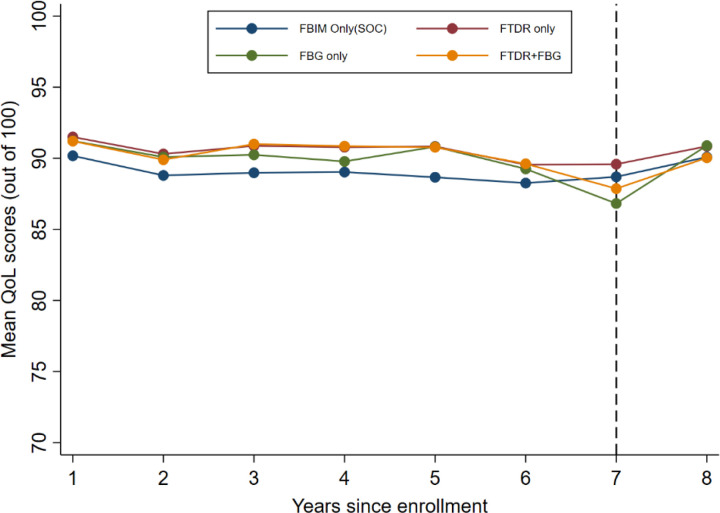
Mean QoL score profile over time by the DSD model categories QoL scores are at 100-percentage points scale. The Y-axis is truncated at 70% for clear visibility of the difference between groups. The dashed black vertical line denotes the 2^nd^ wave 2021 COVID-19 lockdown in Uganda.

At baseline, participants had high QoL scores (≥ 90 percent points) which were sustained above 85 percent points across all comparison groups throughout the 8 years of follow-up. QoL was consistently higher among participants on DSD models compared to the standard of care. QoL declined between years four and seven, particularly among FBG participants and those switching between FBG and FTDR groups, while SOC remained unaffected. This drop recovered by year eight. Participants enrolled in FTDR maintained the highest QoL scores throughout the study period (Fig. 1 and Table 2). A trend test showed a borderline significant linear trend in mean QoL scores (p = 0.052).

### Adjusted analysis comparing mean QoL between the standard of care model and DSD models

Table 3 shows an adjusted comparison of QoL between the comparison group and DSD models. The adjusted mean QoL during the 8 years of follow-up was 3.66 times higher in the DSD group compared to the standard of care group (weighted mean ratio [WMR] 3.66, 95% CI 2.10 to 6.37, p-value < 0.001). Sensitivity analyses using G-computation and traditional regression covariate adjustment approaches yielded similar results. Mean QoL was more than 5 percentage points higher among participants enrolled in the FTDR DSD model compared to the SOC model (WMR 5.47, 95% CI 2.79 to 10.72, p-value < 0.001). However, there was no statistically significant difference in QoL between the FBG model and SOC model (WMR 2.41, 95% CI 0.72 to 8.05, p-value = 0.120), nor for participants who switched between FTDR and FBG models compared to the standard of care model (WMR 2.10. 95% CI 0.93 to 4.71, p-value = 0.073).

### Sustained viral suppression, mortality and Lost to follow-up

Table 4 presents the impact of DSD models on secondary outcomes. DSD models led to higher rates of sustained viral suppression, reduced mortality, and lower risk of LTFU, compared to the standard of care. Participants in DSD models had increased sustained viral suppression compared to those on the standard of care (weighted odds ratio [WOR] 1.69, 95% CI 1.24 to 2.31 at < 200 copies/mL and 1.55, 95% CI 1.09 to 2.21 at < 1000 copies/mL viral load thresholds). Mortality and LTFU were lower in DSD models compared to the standard of care: weighted Hazard Rate (wHR) 0.08, 95% CI 0.03 to 0.20 for mortality and 0.08, 95%CI 0.02 to 0.31 for LTFU. The impact of DSD models on sustained viral suppression at 1000 copies/mL viral load threshold was significantly higher among females but not among males (interaction p-value = 0.03). However, the impact of DSD models on sustained viral suppression did not differ across age groups (interaction p-value = 0.31). Similarly, the effect of the DSD models on mortality did not vary by sex (p = 0.95) or age group (p = 0.92).

## Discussion

This study evaluated the impact of facility-based DSD models on the QoL of PLHIV receiving ART at a large urban HIV clinic in Uganda and to the best of our knowledge, this is the first quasi-experimental study examining this relationship in Uganda. We also evaluated if QoL differed by the duration of care under each model. Our findings indicate that PLHIV enrolled in any DSD model had higher mean QoL scores at baseline and maintained higher mean QoL scores compared to the standard of care throughout the years of follow-up. These findings add to the growing body of evidence showing that PLHIV in DSD models experience higher QoL scores than those in standard of care. A study conducted in Tanzania reported similarly showed higher QoL among stable PLHIV on ART in DSD models compared to the standard of care in cross-sectionally determined scores (19). Our analysis shows that even after almost a decade of follow-up, QoL scores for PLHIV on ART in DSD models remained relatively higher than observed among PLHIV in standard of care.

This may be attributed to convenient ART delivery, reduced stigma, reduced patient expenses related to HIV care, more time to engage in productive work, and high satisfaction associated with DSD models. A study conducted in Nigeria demonstrated high degrees of satisfaction and perceived quality of care with DSD models, potentially influencing QoL (20). QoL was generally highest in the FTDR group while FBG showed no improvement in QoL. These findings suggest that the benefits of DSD on QoL are most pronounced in less intensive models like FTDR, consistent with findings by Mokhele in South Africa, who demonstrated higher levels of client satisfaction among PLHIV enrolled on DSD models, especially those on less intense models (21). Relatedly, prior studies show that FTDR is the most preferred model by PLHIV in urban settings like those in our study (4), perhaps because this model maximizes clinical benefits and QoL. The FBG-only model in our study had more PLHIV with comorbidities of diabetes, hypertension, or cancer than other models, which might explain the relatively lower mean QoL as compared to FTDR.

While QoL scores across all models remained high throughout our study, they generally declined from year 5 reaching until year 7 but fully recovered by year 8. Years 6 and 7 (2020 and 2021) coincided with the COVID-19 pandemic period so findings show that the pandemic negatively impacted QoL among PLHIV. These findings are similar to those from a study conducted in India, which showed that COVID-19 worsened QoL among PLHIV (22). Overall, the COVID-19 effect on the QoL was minimal in the standard of care model. This is likely due to continued access to care among PLHIV in the standard of care model because this model usually optimizes for unstable PLHIV who were likely prioritized during interventions to minimize HIV care disruptions due to COVID-19 lockdowns. Indeed, Izudi (2022) reported that interventions to mitigate COVID-19 disruptions to HIV care prioritized unstable PLHIV (23). Moreover, frequent clinic visits required for standard care clients may have meant that they lived near the facility and, hence were least affected by COVID-19 restrictions. Generally, the mean QoL scores in our study were higher than those recently reported among similar PLHIV populations in sub-Saharan Africa settings using the MOS-HIV tool for QoL measurement. Studies conducted in Uganda, Kenya, and Ethiopia all reported lower QoL scores among PLHIV on ART (24–26). While this difference could be attributed to the effect of DSD models in our study, it may also stem from heterogeneity in participant inclusion criteria, particularly the enrolment of newly initiated and ART-naive PLHIV in the other studies. Consistent with several studies in Subsaharan Africa, our study confirmed better clinical outcomes of higher levels of sustained viral load suppression, all-cause mortality, and retention among PLHIV on DSD models as compared to the SOC (7–9).

## Strengths and limitations

Our study had 82% statistical power in assessing the impact of the DSD model on QoL. We employed a quasi-experimental design using inverse probability treatment weighting, which enabled us to effectively control for confounding factors, thereby attributing observed changes in QoL to DSD models. Furthermore, we analyzed real-world data so the findings have a strong external validity. We employed an adapted version of the Medical Outcomes Study HIV Health Survey (MOS-HIV), a tool that is widely used in HIV studies (27) and validated for use in Uganda (16). Our analysis, applying the new national viral load cut-off (< 200 copies/ml) demonstrated sustained benefits of DSD models, confirming the robustness of the positive impact of DSD models on viral suppression.

Our study is limited by its focus on participants who had been on ART for 10 years. It remains unclear how these findings generalize to PLHIV populations new to ART. We ignored model crossovers lasting less than six months. While six months is a reasonable period to assume the QoL effect of the model, implications of short-term crossovers on the validity of the findings remain unclear. We exclusively examined facility-based DSD models, limiting generalizability to other models. Although benefits observed in less intense models like FTDR may imply potential advantages of community-based approaches, caution is needed in generalizing all findings beyond facility-based settings. Participants in the primary study were conveniently sampled (12), and until 2020, eligibility for enrolment in less intense DSD models like FTDR was restricted to stable clients with a suppressed viral load. As such, the potential for selection bias can not be ruled out although baseline differences were reduced by IPTW. All participants were recruited from a relatively well-resourced HIV clinic in an urban setting limiting the generalizability of finding resource-limited rural ART clinics, where patient experiences could differ.

## Conclusion

We found that DSD models maintained higher QoL among PLHIV. Therefore, health ministries and HIV programs should prioritize enrolling PLHIV on ART into appropriate, less intense DSD models to optimize QoL and well-being. Further, as more PLHIV on ART is enrolled in less intensive DSD models, it is crucial for programs to institutionalize routine QoL assessments. Since QoL measures from the patient’s perspective, aspects of HIV care and health outcomes are not reflected in routine monitoring, measuring it routinely could further optimize the provision of person-centered care. Future research should explore the inclusion of community-based DSD models, using prospective designs and including more diverse PLHIV populations including children, adolescents, and PLHIV newly enrolled in care. This will inform a more comprehensive understanding of the effect of DSD models on QoL.

## Supplementary Material

Supplement 1

## Figures and Tables

**Table 1:** Participants’ socio-demographic and clinical characteristics

Characteristics	FBIM Only (SOC)	FTDR only	FBG only	FTDR + FBG	Total	p-value
	(N = 520)	(N = 326)	(N = 92)	(N = 42)	(N = 980)	
**Male sex, n (%)**	190 (36.5)	123 (37.7)	42 (45.7)	19 (45.2)	374 (38.2)	0.310
**Age, median (IQR)**	45 (40,52)	44 (40,49)	47 (43, 54)	48 (44,51)	45 (40,51)	< 0.001
**Age group, n (%)**						
25–44	243 (46.7)	163 (50.0)	28 (30.4)	11 (26.2)	445 (45.4)	< 0.001
45–54	188 (36.2)	135 (41.4)	43 (46.7)	24 (57.1)	390 (39.8)	
55 and above	89 (17.1)	28 (8.6)	21 (22.8)	7 (16.7)	145 (14.8)	
**Currently has a partner, n (%)**	270 (51.9)	186 (57.1)	53 (57.6)	17 (40.5)	526 (53.7)	0.130
**Employment, n (%)**	415 (80.3)	270 (82.8)	74 (82.2)	34 (81.0)	793 (81.3)	0.823
**Household income (> 30USD), n (%)**	258 (52.5)	172 (55.1)	57 (66.3)	28 (68.3)	515 (55.4)	0.037
**Baseline BMI (kg/m^2^), median (IQR)**	23.0 (20.0, 26.0)	22.0 (20.0, 25.0)	23.0 (21.0, 27.0)	22.5 (19.0, 26.0)	23.0 (20.0, 26.0)	
**BMI categories (kg/m^2^), n (%)**						
<18.5	66 (13.0)	43 (13.3)	6 (6.5)	6 (14.3)	121 (12.6)	0.511
18.5–24.9	270 (53.4)	185 (57.1)	55 (59.8)	21 (50.0)	531 (55.1)	
≥25	170 (33.6)	96 (29.6)	31 (33.7)	15 (35.7)	312 (32.4)	
**Baseline CD4**, Median (IQR)	543 (396, 695)	518 (397, 724)	550 (402, 736)	538 (403, 763)	537 (398, 715)	0.244
**WHO stage 3/4, n (%)**	454 (87.3)	288 (88.3)	81 (88.0)	31 (73.8)	854 (87)	0.067
**Baseline viral load** < 200 copies/mL, **n (%)**	487 (93.8)	314 (96.3)	90 (97.8)	41 (97.6)	932 (95.2)	0.176
**Tobacco use, n (%)**						
Never	406 (78.1)	246 (75.5)	73 (79.3)	33 (78.6)	758 (77.3)	0.777
Past	100 (19.2)	73 (22.4)	18 (19.6)	9 (21.4)	200 (20.4)	
Present	14 (2.7)	7 (2.1)	1 (1.1)	0 (0.0)	22 (2.2)	
**Alcohol use, n (%)**						
Never	136 (26.2)	92 (28.2)	24 (26.1)	9 (21.4)	261 (26.6)	0.284
Past	259 (49.8)	151 (46.3)	51 (55.4)	17 (40.5)	478 (48.8)	
Present	125 (24.0)	83 (25.5)	17 (18.5)	16 (38.1)	241 (24.6)	
**NCDs, n (%)**						
Diabetes	17 (3.3)	5 (1.5)	9 (9.8)	1 (2.4)	32 (3.3)	0.001
Hypertension	120 (23.1)	32 (9.8)	41 (44.6)	17 (40.5)	210 (21.4)	< 0.001
Cancer	2 (0.4)	1 (0.3)	1 (1.1)	0 (0.0)	4 (0.4)	0.728

Note: P-values generated using Kruskal-Wallis test for continuous variables, and chi-square and fishers exact for categorical variables; NCDs: Non-communicable diseases.

**Table 2:** Mean QoL score over time by the DSD model categories

Follow-up period	N	DSD model category					
		Overall Mean (SD)	SOC Mean (SD)	FTDR only Mean (SD)	FBG Mean (SD)	FTDR + FBG Mean (SD)	Any DSD¥
Baseline	980	89.6(7.0)	89.2(7.5)	90.3(6.5)	89.5(6.3)	89.5(6.3)	90.1(6.4)
Year 1	917	90.8(7.3)	90.2(7.9)	91.5(6.3)	91(6.7)	91.2(7.6)	91.4(6.5)
Year 2	965	89.5(8.3)	88.8(9.1)	90.3(7.5)	90.1(7.0)	89.9(6.8)	90.2(7.3)
Year 3	940	89.8(6.6)	89.0(7.6)	90.9(5.3)	90.2(5.6)	91.0(3.9)	90.8(5.3)
Year 4	909	89.8(6.3)	89.0(7.5)	90.8(4.5)	89.8(5.7)	90.9(4.2)	90.6(4.8)
Year 5	522	89.7(6.6)	88.7(8.1)	90.8(4.5)	90.8(4.1)	90.8(4.2)	90.8(4.3)
Year 6	562	88.9(5.7)	88.3(7.0)	89.6(4.1)	89.3(4.0)	89.6(4.1)	89.5(4.0)
Year 7	795	88.8(7.7)	88.7(7.9)	89.6(6.7)	86.8(9.2)	87.9(7.7)	88.8(7.5)
Year 8	763	90.5(7.6)	90.1(7.8)	90.5(7.6)	90.9(7.6)	90.0(6.8)	90.7(7.4)

**QoL** – denotes quality of life, DSD – denotes Differentiated Service Delivery model, SOC – standard of care, FTDR – denotes Fast Track Drug Refill model, FBG – denotes Facility-based Group model, FTDR + FBG – denotes PLHIV who were once on FTDR and switched to FBG model and vice versa.

SD – denotes the standard deviation

¥ Any DSD – includes PLHIV who were on any either FTDR or FBG

† p-value comparing mean QoL scores within each group overtime.

**Table 3:** Weighted mean ratio for QoL scores comparing standard of care model versus DSD models

	Quality of life scores	
	Weighted Mean Ratio (95%CI) †	P-value
**Primary analysis (IPTW)**		
Standard of care	Reference	
Intervention	3.66 (2.10, 6.37)	< 0.001
**Sensitivity analyses (Any DSD versus standard of care)**		
G-Computation (complete cases)	3.05 (2.53, 3.64)	< 0.001
Traditional regression covariate adjustments ††	2.74 (1.62, 4.64)	< 0.001
**Secondary comparisons** ‡		
*Standard of care versus FTDR model*		
Standard of care	Reference	
FTDR	5.47 (2.79, 10.72)	< 0.001
*Standard of care versus FBG models*		
*Standard of care*	Reference	
FBG	2.41 (0.72, 8.05)	0.120
*FBG versus FTDR models*		
FBG	Reference	
FTDR	2.10 (0.93, 4.71)	0.073

QoL denotes quality of life. QoL score was estimated at a 100% scale, SOC – denotes standard of care, DSD – denotes Differentiated Service Delivery model, SD – denotes Standard Deviation, CI – confidence interval, FTDR – denotes Fast Track Drug Refill model, FBG – denotes Facility-based Group model, FTDR + FBG – denotes PLHIV who were once on FTDR and switched to FBG model and vice versa.

‡ Secondary (pairwise comparisons) between DSD models were made by fitting separate models

¥ Any DSD – includes PLHIV who were on any either FTDR or FBG

† Adjusted Mean Ratio in QoL indicates average QoL score change per year among PLHIV on the DSD model compared to those on standard of care model; was estimated using inverse probability treatment weighting (IPTW) weighted mixed effect random intercept model (except the sensitivity analysis using G-computation). Standardized IPTW weights were used in the effect size estimation model. The propensity model and covariates included are presented in Additional file 1 Table S1.

†† Detailed results for traditional regression covariates adjustments are presented in Additional file 1 Table S2

**Table 4:** Sustained viral suppression, mortality, and Lost to follow-up comparing DSD models to the standard of care

Comparisons	*Sustained viral suppression (< 200)* wOR (95%CI) †	*Sustained viral suppression (< 1000)* wOR (95%CI) †	*Mortality* wHR (95%CI)††	*Lost to follow-up* wHR (95%CI)†††
**Primary comparison**				
SOC	1	1	1	1
DSD	1.69 (1.24, 2.31)	1.55 (1.09, 2.21)	0.08 (0.03, 0.20)	0.08 (0.02, 0.31)
**Subgroup** ‡				
**Females**				
SOC	1	1	1	1
DSD	2.11(1.41, 3.16)	2.10(1.32, 3.33)	0.06 (0.02, 0.27)	ND
**Males**				
SOC	1	1	1	1
DSD	1.18 (0.72, 1.94)	0.94 (0.52, 1.67)	0.09 (0.03, 0.30)	ND
*p-value for interaction (Female vs Male)*	0.08	0.03	0.95	
**Age 25–44 years**				
SOC	1	1	1	1
DSD	2.33 (1.47, 3.68)	1.85 (1.12, 3.06)	0.08(0.01, 0.62)	ND
**Age 45 + years**				
SOC	1	1	1	1
DSD	1.27 (0.83, 1.93)	1.28 (0.77, 2.12)	0.07 (0.01, 0.53)	ND
*p-value for interaction (25–44 years vs 45 + years)*	0.06	0.31	0.92	
*TB Negative*				
SOC	1	1	1	1
DSD	1.64 (1.20, 2.24)	1.51 (1.05, 2.16)	0.08 (0.03, 0.20)	ND
*TB positive*				
SOC	1	1	1	1
DSD	5.25 (0.50, 55.00)	3.00 (0.28, 32.24)	3.44e^−15^(4.18e^−14^, 2.84e^−14^)	ND
p-value for interaction (TB Negative vs TB positive)	*0.17*	*0.37*	< 0.01	

SOC – denotes standard of care, DSD – denotes Differentiated Service Delivery model

CI – confidence interval,

ND – denotes not done. Sub-group analyses were not done for lost-to follow-up because of a few events

† wOR denotes the weighted Odds Ratio estimated from inverse probability treatment weighted logistic regression model

†† wHR denotes the weighted Hazard Ratio estimated from inverse probability treatment weighted Cox ‡Proportional hazard regression model. Covariates used to estimate inverse probability treatment weights are presented in the propensity score model in the Supplemental Digital Content Table S1

‡ for subgroup analyses, separate models were fitted with the interaction between the intervention variable and covariate.

Additional file 1 Table S3 shows proportions for each of the above secondary outcomes comparing DSD models to the SOC.

## Abbreviations

ART: Antiretroviral Therapy
CD4: Cluster of Differentiation 4
CCLAD: Community Client-Led ART Delivery
CDDP: Community Drug Distribution Points
DSD: Differentiated Service Delivery
FBG: Facility-Based Grou
FBIM: Facility-Based Individual Management
FTDR: Fast-Track Drug Refill
ICEA: Integrated Clinic Enterprise Application
IRB: Institutional Review Board
LTFU: Loss to Follow-Up
MoH: Ministry of Health
MOS-HIV: Medical Outcomes Study HIV Health Survey
PLHIV: People Living with HIV
QoL: Quality of Life
SMD: Standardized Mean Difference
SOC: Standard of Care
TB: Tuberculosis
WHO: World Health Organization
WMR: Weighted Mean Ratio
IPTW: Inverse Probability of Treatment Weighting
WOR: Weighted Odds Ratio
