# Supplementary material for: Impact of Differentiated Service Delivery Models on Quality of Life among People living with HIV in Uganda – A Quasi-Experimental Study
Source: Res Sq. 2024 Dec 17:rs.3.rs-5443965. Preprint. [Version 1] doi: 10.21203/rs.3.rs-5443965/v1 (PMC11702836; doi:10.21203/rs.3.rs-5443965/v1)
Supplement: Supplement 1 [file NIHPPRS5443965v1-supplement-1.pdf]

Supplementary Files

This is a list of supplementary files associated with this preprint. Click to download.

- NasasiraetalAdditionalFile1.docx
